# Microbial Distribution and Biofilm-Forming Capacity in the Reproductive Tract of Farm Ruminants

**DOI:** 10.3390/ani16010133

**Published:** 2026-01-02

**Authors:** Charinya So-In, Natchaporn Piamalung, Aomsab Kongkaew, Phiyakorn Sriarun, Benyapa Kammungkun, Sawarod Phongchaiwasin, Bongkodkanok Somwaeng, Wichayada Haputon, Thanchanok Wadmuang, Surasak Khankhum, Nuchsupha Sunthamala

**Affiliations:** 1 Department of Veterinary Technology, Faculty of Agricultural Technology, Kalasin University, Kalasin 46000, Thailand; charinya.pi@ksu.ac.th; 2 Department of Biology, Faculty of Science, Mahasarakham University, Mahasarakham 44150, Thailand; 3 Epidemic Simulation and Aetiology Nexus for Infectious Diseases, Mahasarakham University, Mahasarakham 44150, Thailand

**Keywords:** farm ruminant, *H. trogontum*, *B. ovis*, *B.longum*, biofilm genes

## Abstract

Reproductive problems in farm ruminants are often linked to imbalances in the microorganisms living in the reproductive tract and their ability to form biofilms. This study examined the presence of bacteria and their biofilm-forming capacity in cows (*n* = 35), water buffaloes (*n* = 25), and goats (*n* = 33) in Northeastern Thailand. Samples collected from the vulva, urethral opening, and vagina were analyzed using bacterial culture, PCR, and a microtiter biofilm assay. Ten bacterial species were identified. *H. trogontum* and *B. ovis* were most common in water buffaloes and goats, while cows showed higher levels of beneficial bacteria such as *B. longum* and *L. acidophilus*. Biofilm testing showed mostly weak or non-adherent biofilms, with mean absorbance values remaining low across species. Weak biofilms were especially common in goats, whereas cows showed predominantly non-adherent patterns. Biofilm-associated genes (*icaA*, *icaD*, *opp3AB*) were more frequently detected in cows and buffaloes and were moderately correlated with weak biofilm formation. Overall, the results show that each ruminant species has a distinct microbial profile and biofilm behavior within its reproductive tract. These differences may influence susceptibility to reproductive infections and can guide future strategies for improving reproductive health and disease prevention in farm animals.

## Simple Summary

Reproductive health in cows, water buffaloes, and goats is influenced by nutrition, hormones, and the equilibrium of microbes residing within their reproductive systems. Certain bacteria are beneficial and contribute to system health, but others may induce infections resulting in infertility, abortion, or compromised progeny. This study investigated the reproductive tracts of 93 farm animals from Northeastern Thailand to identify the bacterial presence and their capacity to produce biofilms, a protective covering that enhances bacterial survival and treatment resistance. Buffaloes and goats exhibited elevated levels of pathogenic microbes, including *Helicobacter trogontum* (*H. trogontum*) and *Brucella ovis* (*B. ovis*), which may be linked to reproductive complications. Conversely, the cows exhibited a higher prevalence of advantageous bacteria, including *Bifidobacterium longum* (*B. longum*) and *Lactobacillus acidophilus* (*L. acidophilus*), acknowledged for their contribution to maintaining a healthy equilibrium. Most bacteria produced only feeble biofilms, suggesting that they might survive but not successfully resist the body’s innate defenses. These findings may suggest the influence of each animal species’ “microbial fingerprint” on reproductive health. By understanding these bacterial patterns, farmers and veterinarians may adopt more accurate strategies to prevent diseases, improve fertility, and foster sustainable animal production in local agricultural communities.

## 1. Introduction

Reproductive failure in farm ruminants (cows, water buffaloes, and goats), including abortion, stillbirth, preterm delivery, and infertility, may result from various causes, including to several problems, one of which is reproductive tract infections. Reproductive health disorder is influenced by the imbalance of the host’s microbial populations, physiology, and the colonization of the reproductive tract [1]. According to this perspective, reproductive failures constitute a serious One Health issue in addition to being a pertinent economic one [2,3]. The microbiota, which includes both commensal and pathogenic microorganisms, is critical for maintaining homeostasis, regulating fertility, and increasing disease susceptibility. Bacterial biofilm production has emerged as a key element influencing microbial persistence, antibiotic resistance, and host–pathogen dynamics in the ruminants [4]. The vagina of ruminants has a diverse and dynamic microbial community comprising aerobic, facultative anaerobic, and anaerobic bacteria. This complex combination serves as a natural defensive system, inhibiting the unregulated growth of harmful pathogens [5,6]. Notable variation in the dominant colonizing species was observed across studies, highlighting the dynamic nature of the microbial ecosystem. Throughout this ongoing migration, new strains are constantly introduced, adding to microbial diversity. Among the common inhabitants of the cows, water buffaloes, and goats vaginal tract are *Streptococcus* sp., *Staphylococcus* sp., *Enterococci*, and members of *Enterobacteriaceae* [5,6,7,8].

The reproductive system consists of beneficial microorganisms that coexist with pathogenic microorganisms, maintaining a balance within the body, affecting fertility and susceptibility to disease. In addition, bacterial biofilm development is an important factor influencing microbial persistence, bacterial antibiotic resistance, and host–pathogen interactions [9,10]. While biofilms can protect bacterial populations, their presence in the reproductive tract can have both beneficial and detrimental consequences for the cattle, influencing microbial colonization dynamics, persistence, and the outcome of reproductive tract infections. Bacterial biofilms are aggregates of bacteria that attach to a surface and/or to one another, encapsulated in a self-generated matrix. The biofilm matrix comprises molecules such as proteins (e.g., fibrin), polysaccharides (e.g., alginate), and extracellular DNA (eDNA) [11]. Besides the protection provided by the matrix, bacteria in biofilms can utilize several survival tactics to circumvent host defensive mechanisms. By being inactive and concealed from the immune system, they may inflict localized tissue damage and subsequently lead to an acute infection. In the biofilm, bacteria acclimatize to ambient anoxia and nutritional scarcity by demonstrating modified metabolism, gene expression, and protein synthesis, resulting in a diminished metabolic rate and a decreased cell division rate [12]. The creation of biofilms provides a protective barrier to bacteria, shielding them against host immune system attacks and facilitating their colonization and multiplication, eventually promoting the establishment of chronic infections [11,13]. In ruminants, biofilms are best studied in the rumen, where bacteria attach to feed particles and epithelial surfaces and form structured biofilms that are regulated by quorum-sensing systems such as AI-2 signaling [14]. These biofilm–QS interactions influence fermentation patterns, methane production, and feed efficiency. By analogy, biofilm formation in the reproductive tract may similarly modulate microbial persistence, host responses, and fertility outcomes, yet this niche has been far less investigated. Our study therefore aims to characterize site- and species-specific patterns of reproductive tract microbiota, pathogens, and biofilm-forming capacity in farm ruminants.

Due to the critical role of uterine-associated microbiota in reproductive health, physiology, and performance, alterations in microbial composition may significantly influence reproductive outcomes. Currently, it is widely recognized that a healthy uterine microbiota is dependent on bacterial diversity; however, there is still no agreement on the precise composition of this microbiota [15]. While several studies suggest that uterine microbial composition and stability may influence reproductive health, the specific characteristics of a healthy uterine microbiota remain under active investigation. Comprehending the distribution of bacterial infections and microbiota among various ruminant animals is crucial for evaluating possible health hazards and enhancing reproductive management measures. Furthermore, examining the abundance of biofilm-associated genes and their relationship with bacterial species offers insight into the processes underlying biofilm development and its consequences for reproductive health. Despite the growing recognition of biofilm-associated diseases in veterinary medicine, substantial gaps remain in our understanding of species-specific biofilm formation (*icaA*, *icaD*, and *opp3AB* gene) patterns, bacterial co-localization, and the underlying genetic determinants across different anatomical sites within the reproductive tract.

Given the limited understanding of microbial colonization patterns and biofilm-forming capacity within the reproductive tracts of different ruminant species, this study was designed to characterize the distribution of pathogens, commensal microbiota, and biofilm-associated genes across three anatomical sites. We hypothesized that cows, water buffaloes, and goats would exhibit distinct microbial profiles and biofilm-forming behaviors, reflecting species-specific ecological niches within the reproductive tract. Furthermore, we proposed that these differences would correlate with variations in biofilm gene prevalence and phenotypic biofilm formation. Several biofilm-associated genes were assessed in this study due to their established roles in bacterial adherence and biofilm maturation. The *icaA* and *icaD* genes participate in the synthesis of polysaccharide intercellular adhesin, a key component of the extracellular matrix in *Staphylococcus* biofilms. The *opp3AB* operon encodes oligopeptide permease transport proteins involved in nutrient uptake and quorum-sensing pathways that influence biofilm development. The *arcA* gene is part of the arginine deiminase system, supporting bacterial survival under acidic or nutrient-limited conditions encountered within biofilms. The *IS256* insertion sequence has been linked to enhanced genetic adaptability and modulation of biofilm-related phenotypes in several pathogenic species. Together, these genes represent important molecular markers of biofilm-forming potential in diverse reproductive-tract bacteria.

We hypothesized that cows, water buffaloes, and goats would exhibit distinct microbial communities and biofilm-forming capacities across reproductive sites, and that these differences would correlate with variation in biofilm-associated gene profiles. This study aims to investigate the microbial composition and dynamics of biofilm formation in the reproductive systems of farm ruminants, specifically cows, water buffaloes, and goats. It places particular emphasis on the distribution of bacterial pathogens, commensal microbiota, and biofilm-associated genes to enhance our understanding of microbial interactions by examining species-specific colonization patterns and their potential consequences for reproductive health management and prevention.

## 2. Materials and Methods

### 2.1. Experimental Design

Female native Thai cattle, water buffaloes and goats aged 2–3 years from traditionally managed farms in Kalasin Province were included in this study. After 30 days of general observation (no treatment), the experiment was initiated by selecting clinically healthy female native Thai cattle, water buffaloes, and goats. Animals were assessed based on general health status and the absence of apparent reproductive disorders at the time of sampling., 35 of female cows, 25 of female water buffaloes, and 33 of goats for vaginal bacteria collection. All cattle were kept semi-intensive, i.e., from 06:00 PM to 06:59 AM in the barn and from 07:00 AM to 05:59 PM on the floor. The natural diet of Thai native cattle mostly comprises grasses, rice straw, legumes, alfalfa, clover, and hay, as they are predominantly grazing animals [16,17], without control of additional grain and vitamins for individual animals. The climatic conditions for the study were from April 2023 to August 2023, i.e., during the Thai summer season. The study was conducted under the supervision of a veterinarian and in accordance with the proposal (IACUC-MSU-27/2022) approved by the Committee on Ethics and Standards for the Rearing and Use of Animals for Scientific Purposes of Mahasarakham University.

### 2.2. Sample Collection and Bacterial Isolation

**Figure 1:**
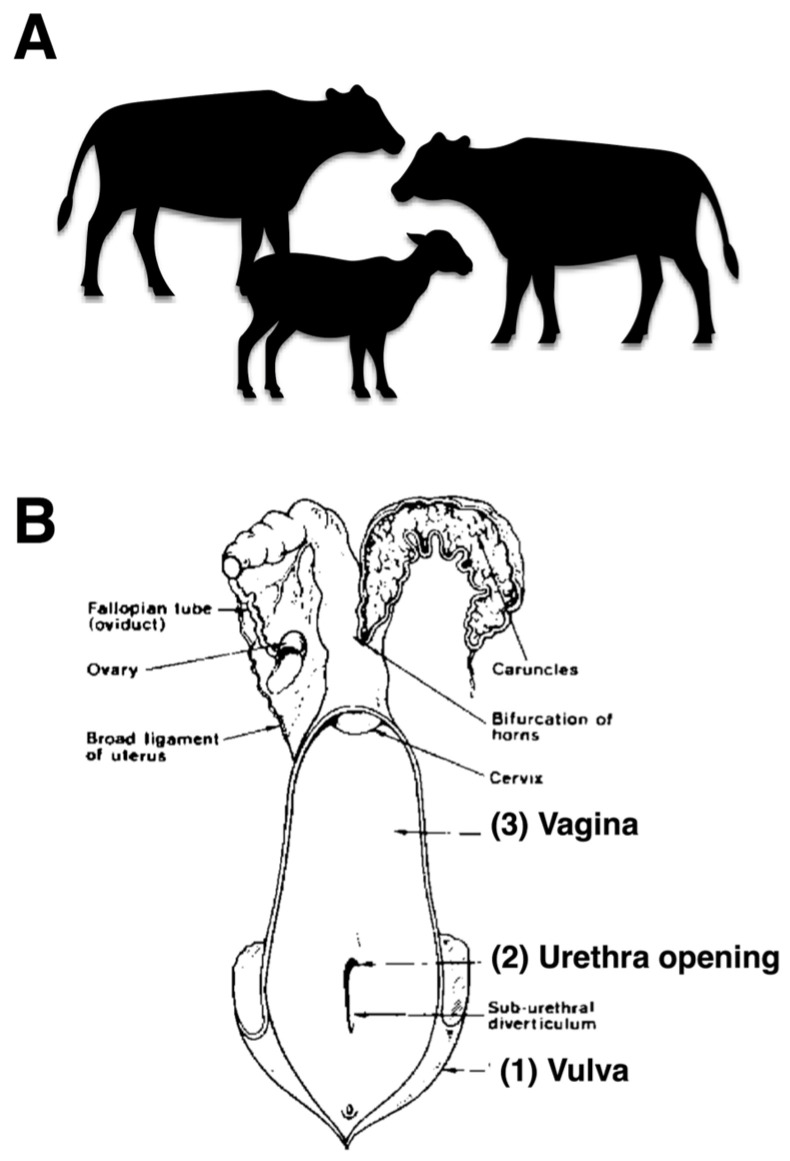
Selecting healthy cattle and sampling sites of the female reproductive tract. (**A**) cows, water buffaloes, and goats (**B**) anatomical diagram illustrating the three sampling sites of the female reproductive tract including vulva, urethral opening, and vagina for bacterial collection.

Bacterial samples were collected from the reproductive tracts of female cattle to investigate bacterial presence. The sampling procedure involved swabbing three specific anatomical sites in the following order: vulva, urethral opening, and vagina, as illustrated in Figure 1A,B. The collected samples were subsequently cultured in Brain Heart Infusion (BHI) broth (Himedia, Mumbai, India) for bacterial isolation. The inoculated broth was incubated at 37 °C for 24 h under aerobic conditions [18]. Estrous cycle stage was not controlled due to field constraints; this is acknowledged as a potential confounder.

### 2.3. DNA Extraction of Vaginal Microorganisms

Genomic DNA was extracted from mixed bacterial cultures using the GF-1 Bacterial DNA Extraction Kit (Vivantis Technologies, Selangor, Malaysia). Approximately 1–3 mL of the 24-h bacterial culture was transferred into a sterile microcentrifuge tube and centrifuged at 6000× *g* for 2 min. The supernatant was discarded, and the pellet was resuspended in 100 µL of Buffer R1. Twenty microliters of lysozyme (1 mg/mL) were used. The mixture was incubated at 37 °C for 20 min. After incubation, the suspension was centrifuged at 10,000× *g* for 3 min. The pellet was resuspended in 180 µL of Buffer R2 and 20 µL of Proteinase K and incubated at 65 °C for 20 min, with gentle mixing every 5 min. Subsequently, twice the volume of Buffer BG (~400 µL without RNase A or ~440 µL with RNase A) was added, and the mixture was gently inverted until homogenous. The sample was incubated at 65 °C for 10 min, followed by the addition of 200 µL of absolute ethanol. The mixture was transferred into a spin column and centrifuged at 10,000× *g* for 1 min. The column was washed with Wash Buffer and centrifuged at 10,000× *g* for 1 min, followed by an additional centrifugation to remove residual ethanol. DNA was eluted using 50–100 µL of pre-warmed Elution Buffer and stored at 4 °C or −20 °C until use [19,20,21,22].

### 2.4. Polymerase Chain Reaction (PCR)

Specific PCR assays were performed to detect target bacterial species using primer pairs listed in Table 1. PCR amplification was carried out in a total reaction volume of 25 µL, containing genomic DNA template, 10X PCR buffer, 50 mM MgCl_2_, forward and reverse primers (10 µM each), 10 mM dNTPs, Taq DNA polymerase, and nuclease-free water (Vivantis Technologies, Selangor, Malaysia). The PCR was conducted using a thermal cycler (Thermo Fisher Scientific, MA, USA) under the following conditions: initial denaturation at 95 °C for 5 min; followed by 40 cycles of denaturation at 95 °C for 15 s, annealing at 50 °C for 30 s (adjusted according to the annealing temperature of each primer pair), and extension at 72 °C for 40 s. A final extension step was performed at 72 °C for 7 min. The amplified PCR products were analyzed by agarose gel electrophoresis (Nippon Genetics EUROPE, Düren, Germany). DNA bands were visualized under UV light using a gel documentation system (Bio-Rad Laboratories, CA, USA), and images were captured for analysis [18]. Because DNA was extracted from mixed microbial samples, PCR detection reflects community-level gene presence rather than species-specific attribution. PCR assays included positive and negative controls in every run, and all primer sets were selected from previously validated studies demonstrating high specificity for the target microbial genes. Although differences were observed among species, the underlying causes—whether ecological, physiological, or management-related—cannot be determined from this study.

### 2.5. Biofilm Formation Assay

Bacterial suspensions were adjusted to a 0.5 McFarland standard. Subsequently, 5 μL of each suspension was inoculated into 100 μL of Tryptic Soy Broth (TSB) (Himedia, Mumbai, India) per well in a 96-well microtiter plate (Thermo Fisher Scientific, MA, USA), and each isolate was tested in triplicate. All isolates had been previously preserved in BHI broth supplemented with glycerol (Himedia, Mumbai, India). The resulting suspensions were adjusted to a 0.5 McFarland standard, and 5 µL of each standardized culture was inoculated into 100 µL of fresh TSB in sterile 96-well microtiter plates. Each sample was analyzed in triplicate. Plates were incubated at 37 °C for 24 and 48 h under static conditions. After incubation, planktonic cells were gently removed, and the wells were washed twice with 200 µL of deionized water to eliminate non-adherent bacteria. The plates were then inverted and allowed to air-dry at room temperature. Biofilms were stained by adding 200 µL of 0.1% (*w*/*v*) crystal violet solution (Sigma-Aldrich, MA, USA) to each well and incubating for 10 min at room temperature. Excess stain was discarded, and the wells were washed twice with deionized water and air-dried. The retained crystal violet was subsequently solubilized with 200 µL of absolute ethanol (Sigma-Aldrich, MA, USA) for 5 min, and the contents were gently mixed by pipetting. A volume of 100 µL from each well was transferred to a new 96-well plate for measurement. Biofilm biomass was quantified by measuring the absorbance at 570 nm using a ELISA Microplate Reader BK-EL 10E (Biobase, Shandong, China). The mean absorbance value for each sample was calculated and normalized relative to the negative control (*Escherichia coli* ATCC 25922). Based on these normalized values, biofilm formation was categorized as non-adherent (<1), weak (1–2), moderate (2–4), or strong (>4), in accordance with previously published criteria [18,19]. Biofilm assays were performed on mixed community cultures derived from reproductive tract swabs. This design was chosen to evaluate the overall biofilm-forming capacity of the community-based microbiome, reflecting the ecological interactions of microorganisms inhabiting the female reproductive tract, rather than biofilm formation by individual isolates [34].

### 2.6. Statistical Analysis

The prevalence of the pathogenic bacteria groups was determined by calculating the ratio of cattle that tested positive for the bacteria to the total number of cattle. Statistical differences in bacteria prevalence were tested using the Chi-square and Fisher’s exact tests in Minitab (version 16.0) software. Prevalence was expressed as a proportion with 95% confidence intervals (95% CI) calculated using the Wilson score method [35]. Correlation analyses were performed using Spearman’s correlation coefficient with two-tailed significance testing. The *p*-values ≤ 0.05 were considered statistically significant, using GraphPad Prism software version 10 [18,19]. Correlation coefficients were interpreted using widely accepted descriptive categories: negligible (0.00–0.10), weak (0.10–0.39), moderate (0.40–0.69), strong (0.70–0.89), and very strong (≥0.90). These thresholds serve as general guides, and we recognize that their exact boundaries may vary among fields and should not be viewed as rigid cutoffs [36].

### 2.7. Limitations Section

Because only aerobic culture was performed, anaerobic or microaerophilic bacteria may be underrepresented. To reduce cross-site contamination, the following measures were implemented: the use of new sterile swabs for each anatomical site, external washing prior to sampling, sampling conducted by a certified veterinarian, and reduced contact between the swab and external tissue. These findings should be interpreted in light of limitations such as culture bias, mixed-DNA PCR detection, and the potential for cross-site contamination inherent in field-based sampling. These limitations should be considered when interpreting the findings; therefore, the results should be viewed as preliminary, forming a basis for more comprehensive, sequencing-based studies in the future

## 3. Results

### 3.1. Distribution of Pathogens and Microbiota

**Figure 2:**
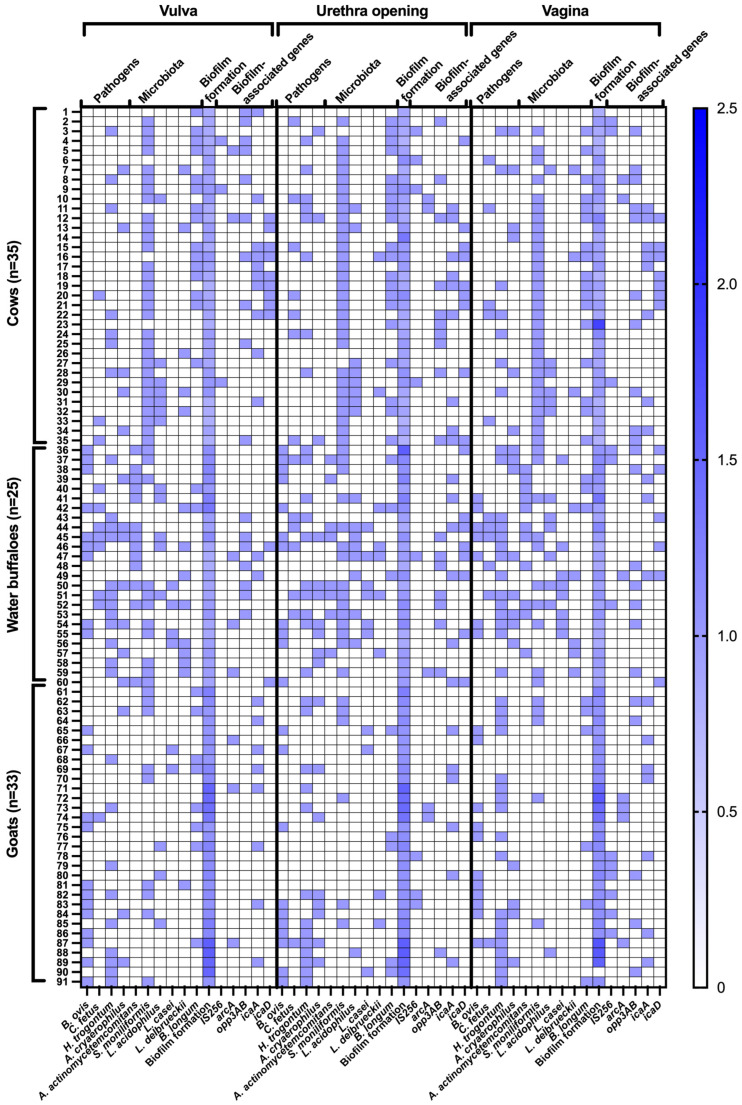
Distribution of detected pathogens and commensal microbiota, biofilm formation patterns, and biofilm-associated genes in the female genital tract of healthy cattle. For microbial detection, blue boxes indicate positive detection, whereas white boxes indicate negative detection. Biofilm-forming capacity is expressed as the ratio of absorbance values of test samples to the negative control, as determined by the microtiter plate assay.

The prevalence of bacterial pathogens and microbiota varies greatly depending on the anatomical location (vulva, urethra, and vagina) in cows, water buffaloes, and goats. *B. ovis* was an extremely rare pathogenic bacterium in cows, however it was found in water buffaloes at a high frequency of 28–44% and in goats at an even higher level of 30.30–36.36% across all locations of collection, with highly significant differences (*p* < 0.000000). Also, *Campylobacter fetus* (*C. fetus*) exhibits a slight incidence in cows (8.57–20%) but was more frequent in buffalo (20–28%). In goats, the occurrence is exceedingly uncommon at 3.03%, demonstrating distinct statistical significance (*p* < 0.001065). *H. trogontum* exhibited the highest prevalence in water buffaloes (40–56%), followed by goats (27.27–54.55%) and cows (20–22.86%), with highly significant interspecies variation (*p* < 0.000059). *A. cryaerophilus* followed a similar trend, with cows (8.57–17.14%) showing lower prevalence compared to water buffaloes (28–32%) and goats (9.09–24.24%), with significant differences (*p* < 0.001402) (Figure 2 and Table 2).

Regarding microbiota composition, *Aggregatibacter actinomycetemcomitans* (*A. actinomycetemcomitans*) was completely absent in cows and goats but was highly prevalent in water buffaloes (32–52%) across all sites, indicating a species-specific colonization pattern (*p* < 0.000000). *Streptobacillus moniliformis* (*S. moniliformis*) was dominant in cows (85.71–88.57%), moderately present in water buffaloes (40–60%), and significantly lower in goats (15.15–27.27%), with extreme statistical significance (*p* < 0.000000). Among lactobacilli, *Lactobacillus acidophilus* (*L. acidophilus*) was detected at relatively stable levels in cows (17.14–22.86%) and water buffaloes (20–32%), whereas it was rare in goats (3.03–9.09%), with significant differences (*p* < 0.000111). *Lactobacillus casei* (*L. casei*) was absent in cows but detected in water buffaloes (16–32%) and at low levels in goats (6.06–9.09%), with strong statistical significance (*p* < 0.000000). Similarly, *Lactobacillus delbrueckii* (*L. delbrueckii*) was present in cows (5.71–14.29%) and more frequently detected in water buffaloes (20–32%), but almost completely absent in goats (*p* < 0.000008). *Bifidobacterium longum* (*B. longum*) showed the highest prevalence in cows (42.86–57.14%), followed by water buffaloes (8–12%) and goats (15.15–24.24%), with statistically significant differences (*p* < 0.000000) see in Table 2. Goats exhibited the lowest bacterial diversity but had a significant presence of *B. ovis*, *H. trogontum*, and *A. cryaerophilus* (Figure 2 and Table 2).

### 3.2. Biofilm Formation Patterns

The prevalence of biofilm-associated genes and biofilm formation patterns varies significantly among cows, water buffaloes, and goats across different anatomical sites (vulva, urethra opening, and vagina). Among biofilm-associated genes, *opp3AB* showed a consistently high prevalence in both cows and water buffaloes (28.57% and 28.00%, respectively) but was absent in goats at the vulva and urethra opening, while present at lower levels in the vagina (15.15%), with significant statistical differences (*p* < 0.000002). The *icaA* gene, responsible for polysaccharide intercellular adhesion, was detected in cows (28.57–22.86%) and goats (24.24–21.21%), but was significantly lower in water buffaloes (8–12%), showing statistical significance (*p* < 0.000693). Similarly, *icaD* was predominantly found in cows (20.00–25.71%) and water buffaloes (8.00–32.00%), but was completely absent in goats, with highly significant differences (*p* < 0.000002). The *IS256* gene, which plays a role in biofilm formation and antibiotic resistance, showed a relatively low presence in cows (8.57–11.43%) and slightly higher levels in water buffaloes and goats, but these differences were not statistically significant. The *arcA* gene, involved in anaerobic metabolism, exhibited a scattered presence across all species with no strong statistical significance.

In terms of biofilm formation patterns, most bacteria in cows exhibited a non-adherent phenotype (91.43–80.00%), while this percentage was significantly lower in water buffaloes (64.00–52.00%), and completely absent in goats (*p* < 0.000000). Weak biofilm formation was highly prevalent in goats (96.97–100.00%) but significantly lower in water buffaloes (36.00–48.00%) and cows (8.57–22.86%), indicating a distinct biofilm-forming ability among species (*p* < 0.000000). Notably, moderate and strong biofilm formation was completely absent across all species and sites, suggesting that the dominant bacterial strains in this study did not develop high-level biofilm structures (Figure 2 and Table 3).

### 3.3. Co-Localization and Correlations of Bacteria and Biofilm-Associated Genes

Weak biofilm formers demonstrated consistent correlations with several biofilm-related genes—particularly *icaA*, *icaD*, and *opp3AB*. In the vulva, *icaA* was present in 45% of weak biofilm samples, while *opp3AB* appeared in 50%. At the urethral orifice, *icaA* and *icaD* were detected in 25% and 30% of samples, respectively. Within the vaginal environment, *opp3AB* (42%) and *icaA* (33%) were the most frequently identified, whereas *arcA* and *icaD* were observed in approximately 28–30% of cases. Notably, the co-localization of *H. trogontum* and *B. longum* with *icaD* was consistently significant across all anatomical sites, suggesting their potential contribution to early biofilm establishment and structural stability. *S. moniliformis* also frequently co-localized with *opp3AB* and *icaA*, implying possible functional interactions in biofilm production (Figure 2 and Table 3).

**Figure 3:**
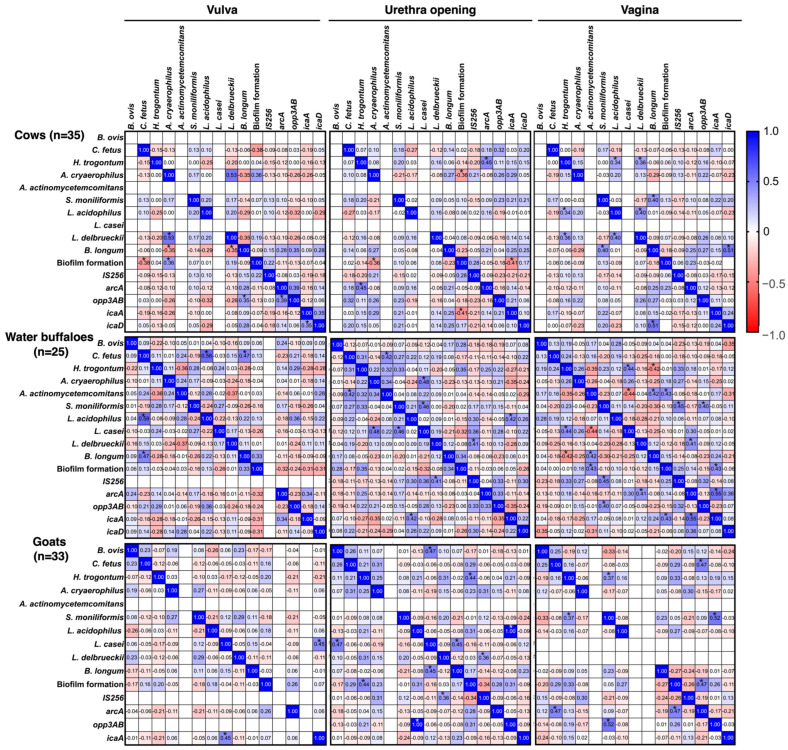
Correlation coefficient analysis of pathogens and microbiota, biofilm formation patterns, and biofilm-associated genes in female genitalia of healthy cattle. * *p* ≤ 0.05.

In the beginning, as shown in Figure 3, positive associations were observed between *opp3AB* and *icaD*, as well as between *icaA* and *B. longum*, within weak biofilm samples. These relationships highlight the cooperative roles of these genes and species in shaping the structural components of biofilms. Likewise, *H. trogontum* showed a strong association with both *icaA* and *opp3AB*, further supporting their collaborative involvement in biofilm initiation. Building upon these findings, the Spearman correlation analysis of biofilm-associated genes and microbial species across the vulva, urethral entrance, and vagina in cows, water buffaloes, and goats revealed distinct site and species-specific interactions influencing biofilm formation. In bovines, biofilm development displayed variable associations depending on the anatomical site. For instance, *B. longum* exhibited a negative correlation with biofilm formation in the vulva (−0.35), whereas *L. delbrueckii* showed a positive relationship (0.53). The genes *opp3AB* and *icaD* also demonstrated meaningful correlations with biofilm development (0.35 and 0.39, respectively). At the urethral entrance, biofilm formation was negatively associated with *C. fetus* (−0.36), while *arcA* exhibited a moderate positive correlation (0.45). Within the vaginal site, *L. acidophilus* (0.34), *L. delbrueckii* (0.36), and *icaA* (0.43) all showed positive correlations with biofilm production.

In water buffaloes, the observed interactions were similarly site-dependent. In the vulva, *L. acidophilus* displayed a moderate positive correlation with biofilm formation (0.58), whereas *B. longum* showed a weaker association (0.47). At the urethral opening, *L. casei* was strongly associated with biofilm formation (0.48), and *opp3AB* exhibited an exceptionally strong correlation (1.00). In the vagina, *opp3AB* (0.52) and *icaA* (0.55) again demonstrated moderate correlations, emphasizing their importance in biofilm development at this location. Fewer notable associations were detected in goats. No significant correlations between bacterial species or genes and biofilm formation were identified in the vulva. At the urethral opening, *B. longum* exhibited a positive association (0.45), while in the vagina, *arcA* (0.47), *opp3AB* (0.52), and *icaA* (0.55) all showed substantial correlations, reinforcing their roles in regulating biofilm formation at this site (Figure 3).

## 4. Discussion

The reproductive microbiome includes bacterial populations found in the vagina, uterus, placental tissues and secretions. Evaluations of the ruminant reproductive microbiome have identified both commensal and pathogenic microorganisms that may affect fertility [9]. This study represents the first report in Thailand to integrate microbial isolation with biofilm formation analysis in the reproductive tracts of farm ruminants. The distribution of bacterial species differed markedly among the vulva, urethra, and vagina of cows, water buffaloes, and goats. We did not directly quantify biofilm matrix components such as exopolysaccharides, proteins, or extracellular DNA; instead, biofilm formation was assessed by microtiter plate assays and biofilm-associated genes. Future work should combine these approaches with targeted assays for matrix components to more precisely link biofilm architecture with reproductive performance. This study provides novel, comparative evidence of how microbial communities and biofilm-associated traits differ among cows, water buffaloes, and goats across distinct reproductive sites. By integrating phenotypic biofilm assays with gene detection and prevalence analysis, the study offers new perspective on the ecological and biological factors shaping reproductive-tract microbiology in ruminants.

In cows, the pathogen with the lowest incidence was *B. ovis*, while the most frequently identified pathogen was *H. trogontum*, responsible for 22.86% of the cases. Approximately 20% of the cases were linked to *C.* fetus, followed by *A. cryaerophilus*, which accounted for 17.14% of the cases. These findings are consistent with previous studies reporting the isolation of *B. ovis*, *C. fetus*, and *A. Cryaerophilus* from the female reproductive tract of cattle with endometritis [37]. The association of these infections, especially *B. ovis* and *A. Cryaerophilus*, with the ability to cause endometritis in cattle, has also been reported [38,39]. While studies investigating these organisms in goats found a low frequency of *C. fetus* (3.03%), the prevalence of *A. cryaerophilus* was comparable to that of cows, with an estimated prevalence of 9.09–24.24%. However, *B. ovis* and *H. trogontum* were more prevalent in goats, accounting for 36.36% and 54.55%, respectively. There were very limited epidemiological studies of *B. ovis* and *H. trogontum* infection in goats in Thailand. These findings are consistent with previous studies of ovine brucellosis in Ethiopia indicating that sheep typically exhibits greater prevalence of Brucellosis than goat [40]. *B. melitensis* is endemic and has a significant detrimental influence on flock productivity in some areas, whereas *B. ovis* is still found in most sheep-raising regions across the world [40,41]. *B. ovis* is a sexually transmitted, infectious illness of domestic sheep characterized by genital lesions and epididymitis in rams, placentitis and uncommon premature births in ewes, and neonatal mortality in lambs. Furthermore, results from Elderbrook et al. [42] study suggest few sheep have been exposed to *B. ovis*, but many flocks contain at least one seropositive animal in Wyoming, USA. *Helicobacter* spp. has been linked to some abortions in sheep from the US, UK, and New Zealand [40]. In a trial involving ewes inoculated with Helicobacter spp. intravenously, 4 ewes aborted or gave birth to weak infected lambs. *H. trogontum* is another bacterium that can cause abortion in sheep. Although there is currently little information on the effect of this infection on abortion in sheep, *H. trogontum* is a common pathogen in New Zealand sheep [43,44,45]. Cows showed a higher frequency of certain commensal species based on culture-dependent methods; however, conclusions regarding microbiota stability cannot be made without sequencing-based analysis.

Overall, these findings suggest that cows harbor a higher prevalence of *icaA* and *icaD*, genes crucial for biofilm formation, particularly in the urethra opening and vulva, while water buffaloes maintain moderate levels. However, despite these genetic markers, actual biofilm formation in cows remains predominantly non-adherent, whereas goats exhibit a significantly stronger tendency toward weak biofilm development [46,47]. The absence of strong biofilm-forming strains across all species could have implications for bacterial persistence and antibiotic resistance, necessitating further research into environmental and genetic factors influencing biofilm phenotypes in different hosts [48,49].

Water buffaloes exhibited a wide range of microbiological features. *B. ovis*, a pathogenic bacterium, was detected at a high frequency of 28–44%. In addition to this organism, a significant number of other pathogens were highly identified, including *C. fetus* (20–28%), *H. trogontum* (40–56%), and *A. cryaerophilus* (28–32%). The incidence of these microbes in buffalo was quite high. This finding is consistent with the results presented by Shi et al. [50], who additionally investigated PubMed, ScienceDirect, SpringerLink, China National Knowledge Infrastructure, Wan fang Database, and VIP Chinese Journal Databases for reports and research studies on the global outbreak of brucellosis in buffalo. Following the findings of this meta-analysis, it was shown that buffalo brucellosis infection has been occurring on a consistent basis in buffalo herds all over the world. Although there are currently no reports of the prevalence of *C. fetus* in water buffaloes, it has been reported in bovine genital campylobacteriosis diagnosis in Spain [51]. In this research, 64 Spanish *C. fetus* field isolates from breeding bulls were identified using phenotypic and PCR techniques, and the results’ consistency was assessed. This sexually transmitted illness causes early reproductive failure, resulting in significant economic losses in the cows sector [52]. Similarly to other organisms not previously reported in buffalo, research has indicated the frequency of *H. trogontum* isolates in pigs, sheep, and mice [43,53], along with the detection of the organism in the bloodstream of individuals exposed to pig excrement on farms. Consequently, *H. trogontum* infection may be categorized as a zoonotic illness [54], and the identification of substantial populations of this bacterium (40–56%) in buffalo underscores the necessity for further investigation in additional animal species. Although some *Campylobacter* and *Helicobacter* species have zoonotic relevance, our findings do not provide evidence of zoonotic transmission [55,56]. The majority of *Arcobacter* isolates were derived from healthy animals, indicating that *Arcobacteraceae* may function as commensals in these hosts. Nevertheless, few instances of Arcobacter isolation from aborted fetuses and mastitis cases have been observed [57]. *A. cryaerophilus* is an emerging worldwide foodborne and zoonotic pathogen. Müller et al. [58] examined 27 *A. cryaerophilus* strains from aquatic fowl in Thuringia, Germany, utilizing whole-genome sequencing. This is consistent with this research which found in buffalo as many as 28–32%. The prevalence of various pathogens in goats revealed that *H. trogontum* was the most common pathogen, at about 27.27–54.55%, followed by *B. ovis* (30.30–36.36%) and *A. cryaerophilus* (9.09–24.24%), while *C. fetus* was very rare in Thai goats, it was uncommon at 3.03%. As with other species, there have been no reports of *H. trogontum*, *B. ovis*, *A. cryaerophilus* and *C. fetus* predominance in goats, particularly Thai goats. The identification of this bacterium within the female reproductive system of goats initiated the investigation of pathogens, including both disease-causing agents and those typically present in goats. The findings indicate that cows harbor a more stable and beneficial microbiota, while water buffaloes are associated with a higher burden of pathogenic bacteria. The presence of *B. ovis*, *H. trogontum*, and *A. cryaerophilus* in goats which could have potential implications for reproductive health. These results highlight host-specific microbial colonization and potential health risks, necessitating further investigation into disease susceptibility and microbiome dynamics among these species.

Animal productivity is affected by reproductive problems that limit fertility. Endometritis and metritis are the most common reproductive diseases in cows, buffalo, and goats, leading to a decrease in lactation. Bacterial infection is a prevalent cause of uterine inflammation, which may arise during or immediately after parturition, coitus, or artificial insemination. Various dangerous microorganisms have been linked to infections of the animal reproductive system [47]. An intrauterine infusion or the systemic administration of antibiotics are the most frequently employed treatments for reproductive tract infections. Nevertheless, antibiotic resistance is the result of the improper use of antimicrobials, which is attributed to three fundamental types of mechanisms: intrinsic, acquired, and adaptive resistance. The formation of biofilm is particularly important in the adaptive resistance of microorganisms [47,59,60]. The *opp3AB* gene, identified as a biofilm-associated gene, was continuously detected in the vulvar and urethral openings of cows and water buffaloes, with prevalence rates of 28.57% and 28.00%, respectively. However, this pattern was not observed in goats from the same geographical area, indicating species-specific differences in microbial distribution. The expression of this gene was considerable in the area below the vagina (between the vestibule and vulva), observed in 15.15% of subjects. The *opp3AB* gene is a biofilm-associated gene discovered in the arginine catabolic mobile element (ACME) (particularly the opp3-operon), which is prevalent in staphylococci such as *S. epidermidis* and *S. aureus* [61]. The film encodes a putative oligopeptide permease structure which supports staphylococci colonization and survival [19,62].

The *icaA* gene associated with polysaccharide intercellular adhesion was identified in cows (28.57–22.86%) and goats (24.24–21.21%) but was markedly reduced in water buffaloes (8–12%). *S. aureus* biofilms enable bacterial adherence to varied substrates and make therapeutic intervention difficult. The *icaABCD* operon and the *icaA* and *icaD* genes are crucial to biofilm production that produces the biofilm-forming genes *icaA*, *icaD*, *icaB*, and *icaC*. The *icaA* and *icaD* genes are key to exopolysaccharide production [63]. The transferase-active *icaA* protein is produced by the *icaA* gene. The *icaA* protein alone has little N-acetyl glucosaminyl transferase activity. Concurrent expression of *icaA* and *icaD* increases transferase activity by 20-fold, resulting in *icaA* and *icaD* protein synthesis [64]. Clinically, bacterial biofilms are a growing problem. Over 65% of clinical infections involve biofilms [65]. According to our research, *icaA* and *icaD* genes were detected in cows, water buffaloes and goats with highly significant differences (*p* < 0.000002). While the *IS256* gene, which is involved in biofilm formation and antibiotic resistance, was low in cows (8.57–11.43%) and slightly higher in water buffaloes and goats, but the differences were not statistically significant. The anaerobic metabolism gene *arcA* was identified in all species but not statistically significant.

Genetic components like insertion sequences (IS) may influence phenotypic diversity in *S. epidermidis*. Notably, *IS256*, which is a mobile genetic element found in several chromosomal copies, has been linked to virulence, presumably via the production of a transposase. *IS256* can influence *ica* gene expression, potentially leading to a biofilm-negative phenotype [62,66]. However, in this research, *IS256*, the identification of this gene may not be statistically significant. This aligns with previous studies, which also may contribute to virulence through alternative mechanisms. Indeed, *IS256* is considered a marker of pathogenicity and genomic plasticity in S. epidermidis. There is increasing evidence that changes in gene expression during initial bacterial adhesion and intercellular adhesion (biofilm formation) offer promising avenues for new therapeutic interventions targeting *S. aureus* and *S. epidermidis* biofilm formation, presenting potentially advantageous alternatives to existing treatments [67].

When illustrating the associations between biofilm-associated genes and the microbiome in this study, positive correlations were identified between *opp3AB* and *icaD*, as well as between *icaA* and *B. longum*, in weak biofilm-forming samples. These correlations suggest that the coordinated expression of key biofilm-related genes may facilitate microbial persistence and biofilm development. Furthermore, the co-occurrence of these genes with both commensal and potentially pathogenic bacteria indicates that biofilm formation may support bacterial survival and colonization within the reproductive tract, thereby influencing host–microbe interactions and potentially contributing to the persistence of opportunistic pathogens. These findings emphasized the collaborative function of these genes in the establishment of biofilm structure. The association between genes and the genital microbiome is weak as the microbiome is affected by numerous factors outside genetics, including hormones, environment, hygiene, health status and behavior [33,68]. Although host genetics contribute to microbial composition, the relationship between them is complicated and under ongoing investigation, with environmental and physiological factors frequently exerting a more substantial and immediate influence [62,69]. Previous studies indicate that biofilm formation in ruminant-associated microbiota is largely regulated by quorum sensing, which coordinates microbial aggregation, stress tolerance, and metabolic cooperation at the community level rather than through single structural genes. Within this framework, regulatory genes such as *ftsH*, encoding an ATP-dependent protease involved in protein quality control and stress adaptation, may indirectly support biofilm-associated or biofilm-prone lifestyles by enhancing bacterial resilience under host-associated conditions. Experimental studies using quorum-sensing–modulating compounds, including quercetin and furanone derivatives, further demonstrate that altering microbial communication can reshape microbial structure and stability, supporting a community-level interpretation of biofilm-related markers rather than direct matrix-driven biofilm formation [20,21,22].

These findings highlight the cooperative function of these genes in biofilm structure creation. Similarly, *H. trogontum* revealed a high connection with *icaA* and *opp3AB*, suggesting a synergistic function in biofilm initiation. While studies on *Staphylococcus* species show a strong correlation, specific research on *H. trogontum* and the *ica* genes is limited. It is important to note that other mechanisms for biofilm formation exist and might be more significant in *H. trogontum*. Future research should focus on the role of the *ica* genes in *H. trogontum* and its potential as a biofilm-related virulence factor in *H. trogontum* infections. The Spearman correlation study of biofilm-associated genes and microbial species across various anatomical sites (vulva, urethral entrance, and vagina) in cows, water buffaloes, and goats reveals distinctive interactions that influence biofilm formation based on site and species. In bovines, biofilm development exhibited diverse associations with microbial species and genes across different anatomical locations.

This study suggests that in the vulva, *B. longum* exhibited an adverse correlation with biofilm formation, implying that its presence is associated with a reduction or lack of biofilm development [70,71]. While, *L. delbrueckii* exhibited a positive correlation of 0.53. The biofilm-associated genes *opp3AB* and *icaD* exhibited significant relationships with biofilm development, with values of 0.35 and 0.39, respectively. At the urethral opening, biofilm formation exhibited a negative association with *C. fetus* (−0.36), whereas the gene *arcA* demonstrated a moderate positive correlation (0.45). *L. acidophilus* (0.34) and *L. delbrueckii* (0.36) had a positive association with biofilm production in the vagina, while *icaA* demonstrated a positive correlation of 0.43. This constitutes the inaugural report of such a relationship in animals. Water buffaloes as well as exhibited species and site-specific correlations. In the vulva, *L. acidophilus* was modestly linked with biofilm production (0.58), whereas *B. longum* had a lesser positive correlation (0.47). At the urethral opening, *L. casei* had a substantial positive connection with biofilm formation (0.48), as did the gene *opp3AB* (1.00). In the vagina, *opp3AB* (0.52) and *icaA* (0.55) had a moderate correlation with biofilm production, indicating a critical function in biofilm development. In goats, fewer relationships were seen. There were no significant connections between microbial species or genes and biofilm production in the vulva. At the urethral entrance, B. longum had a positive connection with biofilm formation (0.45). In the vagina, *arcA* (0.47), *opp3AB* (0.52), and *icaA* (0.55) were substantially linked with biofilm development, showing their role in biofilm control at this site [72,73].

## 5. Conclusions

The findings of this study demonstrated the complexity of the interactions between microbes and genetics that are responsible for the production of biofilm in the reproductive tract. The presence of *icaA*, *opp3AB*, and *icaD* was strongly associated with weak biofilm formation, while *H. trogontum*, *B. longum*, and *S. moniliformis* showed significant co-occurrence with these genes, suggesting their involvement in biofilm development and persistence. In addition to providing useful insights into the microbial ecology of biofilm formation, our findings also reveal potential targets for the management of biofilm-associated illnesses in various anatomical regions.

## Figures and Tables

**Table 1:** Specific primers for detection of bacteria pathogens, microbiota, and biofilm-associated genes by polymerase chain reaction.

Category	Organisms and Primers	Annealing Temperature (°C)	PCR Product Size (Base Pair)	References
Pathogenic bacteria strains	*Brucella ovis*	55	228	[23]
	FW: GCCTACGCTGAAACTTGCTTTTG RV: ATCCCCCCATCACCATAACCGAAG			
	*Campylobacter fetus*	55	485	[24]
	FW: AACGACAAATGTAAGCACTC RV: TATTTATGCAAGTCGTGCGA			
	*Helicobacter trogontum*	50	887	[25]
	FW: CATAGGTAACATGCCCCA RV: CTGTTTCAAGCTCCCC			
	*Arcobacter cryaerophilus*	50	255	[26]
	FW: TGCTGGAGCGGATAGAAGTA RV: AACAACCTACGTCCTTCGAC			
Microbiota bacteria strains	*Aggregatibacter actinomycetemcomitans*	58	194	[27]
	FW: ATTGGGGTTTAGCCCTGGT RV: GGCACAAACCCATCTCTGA			
	*Streptobacillus moniliformis*	57	296	[28]
	FW: GCTTAACACATGCAAATCTAT RV: AGTAAGGGCCGTATCTCA			
	*Lactobacillus acidophilus*	57	397	[29]
	FW: GGAAGCTCAAGACCAAATCATG RV: CTTCTTCAAAACATAAACTTGTG			
	*Lactobacillus casei*	60	202	[29]
	FW: ATCATGGAATTGATGGATACCA RV: TAGACTTGATAACATCTGGCTT			
	*Lactobacillus delbrueckii*	65	230	[29]
	FW: TACTGTTAAGGTTGGCGACAGC RV: TGTAGACTTGGCCCTTGAAAGT			
	*Bifidobacterium longum*	57	161	[29]
	FW: GTATCCGTCCGACCCAGCAG RV: GGTGACGGAGCCCGGCTTG			
Biofilm associated genes	*IS256*	55	1102	[30,31]
	FW: TGAAAAGCGAAGAGATTCAAAGC RV: ATGTAGGTCCATAAGAACGGC			
	*arcA*	51	1942	[32]
	FW: CTAACACTGAACCCCAATG RV: GAGCCAGAAGTACGCGAG			
	*Opp3AB*	52	1183	[32]
	FW: GCAAATCTGTAAATGGTCTGTTC RV: GAAGATTGGCAGCACAAAGTG			
	*icaA*	54	188	[33]
	FW: TCTCTTGCAGGAGCAATCAA RV: TCAGGCACTAACATCCAGCA			
	*icaD*	50	198	[33]
	FW: ATGGTCAAGCCCAGACAGAG RV: CGTGTTTTCAACATTTAATGCAA			

**Table 2:** Prevalence of bacterial pathogens and microbiota in female genitalia of healthy cattle.

Collection Sites												
Cattle	Vulva				Urethra Opening				Vagina			
	Cows (*n* = 35)	Water Buffaloes (*n* = 25)	Goats (*n* = 33)	Fisher’s Exact Test (*p* ≤ 0.05)	Cows (*n* = 35)	Water Buffaloes (*n* = 25)	Goats (*n* = 33)	Fisher’s Exact Test (*p* ≤ 0.05)	Cows (*n* = 35)	Water Buffaloes (*n* = 25)	Goats (*n* = 33)	Fisher’s Exact Test (*p* ≤ 0.05)
Pathogens (%) [95% CI]												
*B. ovis*	0.00 (0–9.93)	36.00 (20.16–55.53)	36.36 (21.35–54.22)	0.000000	0.00 (0–9.89)	44.00 (26.67–62.93)	30.30 (17.38–47.34)	0.000000	0.00 (0–9.89)	28.00 (14.28–47.58)	33.33 (19.08–51.50)	0.000000
*C. fetus*	8.57 (2.96–22.34)	28.00 (14.11–47.18)	3.03 (0.54–15.46)	0.000000	20.00 (10.04–35.89)	24.00 (11.50–43.43)	3.03 (0.54–15.32)	0.000085	14.29 (6.27–29.91)	20.00 (8.86–39.13)	3.03 (0.54–15.32)	0.001065
*H. trogontum*	20.00 (10–35.6)	48.00 (30–66.3)	27.27 (14.56–45.21)	0.000059	22.86 (12.07–39.02)	40.00 (23.40–59.26)	39.39 (24.68–56.32)	0.014846	20.00 (10.04–35.89)	56.00 (36.77–73.31)	54.55 (37.24–70.96)	0.000000
*A. cryaerophilus*	14.29 (6.27–29.91)	28.00 (14.11–47.18)	9.09 (3.16–23.74)	0.001201	8.57 (2.96–22.38)	28.00 (14.28–47.58)	24.24 (12.83–41.02)	0.001402	17.14 (8.33–31.72)	32.00 (17.20–51.59)	12.12 (5.24–25.51)	0.001395
Microbiota (%) [95% CI]												
*A. actinomycetemcomitans*	0.00 (0–9.93)	52.00 (33.5–70)	0.00 (0–10.45)	0.000000	0.00 (0–9.89)	32.00 (17.2–51.6)	0.00 (0–10.43)	0.000000	0.00 (0–9.89)	44.00 (26.67–62.93)	0.00 (0–10.43)	0.000000
*S. moniliformis*	85.71 (70.74–93.82)	44.00 (25.6–63.9)	27.27 (14.56–45.21)	0.000000	88.57 (74.05–95.46)	60.00 (40.74–76.6)	18.18 (8.61–34.39)	0.000000	85.71 (70.74–93.82)	40.00 (23.40–59.26)	15.15 (6.65–30.92)	0.000000
*L. acidophilus*	20.00 (10–35.6)	20.00 (9.45–37.73)	9.09 (3.16–23.74)	0.055023	22.86 (12.07–39.02)	32.00 (17.2–51.6)	3.03 (0.54–15.32)	0.000001	17.14 (8.33–31.72)	24.00 (11.50–43.43)	3.03 (0.54–15.32)	0.000111
*L. casei*	0.00 (0–9.93)	16.00 (6.37–34.74)	6.06 (1.69–19.57)	0.000069	0.00 (0–9.89)	24.00 (11.50–43.43)	9.09 (3.14–23.57)	0.000000	0.00 (0–9.89)	32.00 (17.20–51.59)	0.00 (0–10.43)	0.000000
*L. delbrueckii*	14.29 (6.27–29.91)	32.00 (16.65–51.98)	3.03 (0.54–15.46)	0.000000	5.71 (1.58–18.61)	20.00 (8.86–39.13)	6.06 (1.68–19.61)	0.000898	8.57 (2.96–22.38)	20.00 (8.86–39.13)	0.00 (0–10.43)	0.000008
*B. longum*	42.86 (27.33–59.55)	8.00 (2.22–25.00)	24.24 (12.36–41.92)	0.000000	57.14 (40.86–72.02)	12.00 (4.17–29.96)	15.15 (6.65–30.92)	0.000000	48.57 (32.26–65.20)	12.00 (4.17–29.96)	18.18 (8.61–34.39)	0.000000

**Table 3:** Percentage of biofilm associated genes and biofilm formation pattern of bacterial in female genitalia of healthy cattle.

Collection Sites												
Cattle	Vulva				Urethra Opening				Vagina			
	Cows (*n* = 35)	Water Buffaloes (*n* = 25)	Goats (*n* = 33)	Fisher’s Exact Test (*p* ≤ 0.05)	Cows (*n* = 35)	Water Buffaloes (*n* = 25)	Goats (*n* = 33)	Fisher’s Exact Test (*p* ≤ 0.05)	Cows (*n* = 35)	Water Buffaloes (*n* = 25)	Goats (*n* = 33)	Fisher’s Exact Test (*p* ≤ 0.05)
Biofilm associated genes (%) [95% CI]												
*IS256*	8.57 (2.96–22.34)	0.00 (0–13.3)	0.00 (0–10.2)	0.481901	11.43 (4.53–25.6)	4.00 (0.7–19.5)	9.09 (3.16–23.7)	0.145876	8.57 (2.96–22.34)	12.00 (4.25–29.9)	18.18 (9.0–33.7)	0.121238
*arcA*	5.71 (1.58–18.6)	12.00 (4.25–29.9)	9.09 (3.16–23.7)	0.293758	5.71 (1.58–18.6)	4.00 (0.7–19.5)	3.03 (0.54–15.5)	0.965605	5.71 (1.58–18.6)	4.00 (0.7–19.5)	9.09 (3.16–23.7)	0.319819
*opp3AB*	28.57 (16.6–44.8)	28.00 (14.1–47.2)	0.00 (0–10.2)	0.000000	28.57 (16.6–44.8)	28.00 (14.1–47.2)	3.03 (0.54–15.5)	0.000002	28.57 (16.6–44.8)	28.00 (14.1–47.2)	15.15 (7.2–29.4)	0.042214
*icaA*	28.57 (16.6–44.8)	8.00 (2.2–25)	24.24 (13–40.6)	0.000693	25.71 (14.6–41.5)	24.00 (11.3–42.5)	18.18 (9.0–33.7)	0.410656	22.86 (12–38.5)	12.00 (4.25–29.9)	21.21 (11–37.4)	0.104874
*icaD*	25.71 (14.6–41.5)	8.00 (2.2–25)	0.00 (0–10.2)	0.000000	25.71 (14.6–41.5)	32.00 (17–51)	0.00 (0–10.2)	0.000000	20.00 (10–35.6)	24.00 (11.3–42.5)	0.00 (0–10.2)	0.000002
Biofilm-forming capacity (%) [95% CI]												
Non-adherant	91.43 (77–97)	64.00 (44–80)	0.00 (0–10.2)	0.000000	77.14 (61–88)	52.00 (33–70)	0.00 (0–10.2)	0.000000	80.00 (64–90)	64.00 (44–80)	0.00 (0–10.2)	0.000000
Weak	8.57 (2.9–22.3)	36.00 (20–55)	100.00 (89–100)	0.000000	22.86 (12–38.5)	48.00 (30–66)	100.00 (89–100)	0.000000	20.00 (0–10.2)	36.00 (10–36)	96.97 (20–55)	0.000000
Moderate	0.00 (0–9.9)	0.00 (0–13.3)	0.00 (0–10.2)	ns	0.00 (0–9.9)	0.00 (0–13.3)	0.00 (0–10.2)	ns	0.00 (85–99.8)	0.00 (0–9.9)	3.03 (0–15.5)	ns
Strong	0.00 (0–9.9)	0.00 (0–13.3)	0.00 (0–10.2)	ns	0.00 (0–9.9)	0.00 (0–13.3)	0.00 (0–10.2)	ns	0.00 (0–9.9)	0.00 (0–13.3)	0.00 (0–10.2)	ns

Note: ns: no statistic significant.

## Data Availability

All data generated or analyzed during this study are included in the article and are available from the corresponding author upon reasonable request.
